# Prevalence and socioeconomic burden of diabetes mellitus in South Korean adults: a population-based study using administrative data

**DOI:** 10.1186/s12889-021-10450-3

**Published:** 2021-03-20

**Authors:** Sung-Hee Oh, Hyemin Ku, Kang Seo Park

**Affiliations:** 1 College of Pharmacy and Research Institute of Pharmaceutical Sciences, Kyungpook National University, Daehak-ro 80, Buk-gu, Daegu, 41566 Republic of Korea; 2 NDnex, Saebitgongwon-ro 67, Gwangmyeong-si, Gyeonggi-do 14348 Republic of Korea; 3 Division of Endocrinology and Metabolism, Department of Internal Medicine, Deajeon Eulji Medical Center, Eulji University, Dusanseo-ro 95, Seo-gu, Daejeon, 35233 Republic of Korea

**Keywords:** Diabetes mellitus, Prevalence, Economic burden, South Korea, Cost-of-illness

## Abstract

**Background:**

Diabetes leads to severe complications and imposes health and financial burdens on the society. However, currently existing domestic public health studies of diabetes in South Korea mainly focus on prevalence, and data on the nationwide burden of diabetes in South Korea are lacking. The study aimed to estimate the prevalence and economic burden of diabetes imposed on the South Korean society.

**Methods:**

A prevalence-based cost-of-illness study was conducted using the Korean national claims database. Adult diabetic patients were defined as those aged ≥20 years with claim records containing diagnostic codes for diabetes (E10-E14) during at least two outpatient visits or one hospitalization. Direct costs included medical costs for the diagnosis and treatment of diabetes and transportation costs. Indirect costs included productivity loss costs due to morbidity and premature death and caregivers’ costs. Subgroup analyses were conducted according to the type of diabetes, age (< 65 vs. ≥65), diabetes medication, experience of hospitalization, and presence of diabetic complications or related comorbidities.

**Results:**

A total of 4,472,133 patients were diagnosed with diabetes in Korea in 2017. The average annual prevalence of diabetes was estimated at 10.7%. The diabetes-related economic burden was USD 18,293 million, with an average per capita cost of USD 4090 in 2019. Medical costs accounted for the biggest portion of the total cost (69.5%), followed by productivity loss costs (17.9%), caregivers’ costs (10.2%), and transportation costs (2.4%). According to subgroup analyses, type 2 diabetes, presence of diabetic complications or related comorbidities, diabetes medication, and hospitalization represented the biggest portion of the economic burden for diabetes. As the number of complications increased from one to three or more, the per capita cost increased from USD 3991 to USD 11,965. In inpatient settings, the per capita cost was ~ 10.8 times higher than that of outpatient settings.

**Conclusions:**

South Korea has a slightly high prevalence and economic burden of diabetes. These findings highlight the need for effective strategies to manage diabetic patients and suggest that policy makers allocate more health care resources to diabetes. This is the first study on this topic, conducted using a nationally representative claims database in South Korea.

**Supplementary Information:**

The online version contains supplementary material available at 10.1186/s12889-021-10450-3.

## Background

Diabetes mellitus is widely prevalent and imposes a substantial socioeconomic burden on individuals and the society in terms of large medical costs [1, 2]. According to a global report on diabetes, published by the World Health Organization, an estimated 422 million adults were living with diabetes in 2014, compared with 108 million adults in 1980. The worldwide prevalence of diabetes has nearly doubled since 1980, growing from 4.7 to 8.5% in the adult population [3]. Based on cost estimates from a recent systematic review [3, 4], the estimated total global cost of diabetes is more than USD 827 billion in 2014. The International Diabetes Federation (IDF) revealed that the total costs had more than tripled during 2003–2013, with associated increases in the prevalence of diabetes and per capita diabetes cost. The prevalence and the economic burden of diabetes are expected to continue to increase [5, 6].

Diabetes also causes suffering in humans. It leads to complications such as blindness, amputation, renal disease, and cardiovascular disease, which have become major causes of productivity loss due to morbidities and premature mortality [3]. These complications are associated with increased healthcare utilization such as having more outpatient visits and a higher probability of being hospitalized, and with increased use of more medications [7, 8]. Therefore, complications associated with diabetes drive the escalating costs of diabetes management and their financial burden on the healthcare system.

Together with prevalence, morbidity, and mortality data, cost-of-illness (COI) studies have been conducted to measure the economic burden of diabetes in several countries [1, 9]. However, existing domestic public health studies of diabetes in South Korea mainly focus on diabetes prevalence, and data on the nationwide economic burden of diabetes in Korea are lacking. The prevalence of diabetes among Korean adults has increased from 1.5% in 1971 to 9.9% in 2009, and is expected to increase more over time with ageing of population, increasing obesity, and improved longevity [10]. In addition to increasing the number of patients with diabetes, there will be changes in demographics (age, sex ratios) and medical fees [11], which will affect the economic burden of diabetes as time progresses. To address these concerns, this study aimed to estimate the prevalence and the economic burden of diabetes at the population levels in 2019 by using nationally representative claims records that encompass the entire Korean population. Identification of the nationwide burden of diabetes and the patient subgroups with a higher contribution to economic costs can aid in planning prevention and management strategies.

## Methods

### Study design and study population

This study was approved by the Institutional Review Board at the Korea National Institute for Bioethics Policy (P01–201909–21-010). This COI study was conducted using a prevalence-based approach to quantify the economic burden of diabetes at a given point in time [2]. The study estimates the annual cost of diabetes during 1 year for the prevalence cohort of individuals with diabetes. The Health Insurance Review and Assessment Service National Patient Sample (HIRA-NPS) was used for this study. The sample data included approximately 1,450,000 individuals who comprised a random selection of 3% of the entire Korean population covered under the mandatory National Health Insurance or Medical Aid system in 2017. This claims data provided various information: the general information such as gender, age, and indicators for inpatient/outpatient services; healthcare service information including procedures, prescribed drugs, and treatments; and the diagnostic information using the International Classification of Diseases, 10th revision (ICD-10) code [12].

Patients with diabetes were identified using a validated administrative data algorithm [13]. We included adult patients aged ≥20 years and who had claim records containing the diagnostic code for diabetes in at least two outpatient visits or one hospitalization between January 1, 2017, and December 31, 2017. Based on the literature review [13, 14], the diagnostic codes for diabetes were identified as E10 (type1 diabetes mellitus), E11 (type2 diabetes mellitus), E12 (malnutrition-related diabetes mellitus), E13 (other specified diabetes mellitus), and E14 (unspecified diabetes mellitus) as listed in the International Classification of Diseases-10th Revision (ICD-10 codes).

### Estimating the economic burden of diabetes

The economic burden of diabetes was estimated from the perspective of society. The study included both direct and indirect costs. Direct costs reflected the resources used in treating the disease, including expenditure for the diagnosis and treatment of diabetes as medical costs and expenditure for transportation to medical visits as nonmedical costs. Medical costs covered by insurance were estimated using the HIRA-NPS claims data, and medical costs not covered by insurance were calculated using the ratio of out-of-pocket expense to payment for covered services (for which we used the value of 0.25) obtained from data published by the National Health Insurance Service [15]. Transportation unit costs for patients were calculated using data from the Third Korea National Health and Nutrition Examination Survey [16]. Transportation costs were calculated by multiplying round trip unit cost with the number of visits per patient and the total number of diabetic patients. Indirect costs represented the present and future resources lost by patients and their families because of the disease. These costs included the societal costs of caregivers’ time costs, and productivity loss due to morbidity and premature mortality. The caregiver’s cost was calculated as the product of the average annual inpatient days per patient due to diabetes and the average market price for the daily charge of a caregiver, and then it was multiplied by care rate (inpatients that employed a caregiver/total inpatients). The productivity loss cost due to morbidity means the opportunity costs of time lost because of hospitalization or outpatient visits for diabetes treatment. Based on the human capital approach, which estimates the lost productivity as the expected earnings lost due to a disease [17], it was calculated by multiplying the number of visits per patient with age- and gender- specific average daily incomes and employment rates for the age of 20–65 years. The productivity loss cost due to premature mortality was estimated as losses of potential earnings until the age of 65 because of premature death caused by diabetes. It was calculated by multiplying the number of diabetes-related deaths in 2017 with age- and gender- specific average annual incomes and employment rates for each deceased person. A discount rate of 5% was applied to reflect the present value of future incomes. The age- and gender-specific numbers of deaths attributable to diabetes were obtained from the HIRA-NPS claims data. Average daily incomes were derived from the age- and gender-specific average annual incomes provided by the Korean Statistical Information Service [18]. Variables, data sources, and calculation methods used in cost estimation for each cost component are presented in Table 1.

**Table 1:** Variables, data sources, and calculation methods by cost component

Cost component	Variable	Description	Value	Calculation method	Sources
Direct cost					
Medical cost					
	Total cost covered by insurance	Total annual cost covered by insurance in 2017, calculated by target groups	10,120 million USD (in base-case)	A	1)
	Ratio of out-of-pocket expense to payment for covered services	To estimate cost not covered by insurance	0.248	B	2)
	Consumer Price Index	On healthcare component	1.006	C	3)
	Total medical cost	Total annual cost including cost covered and not covered by insurance in 2019	12,710 million USD (in base-case)	D = A × B × C	
Nonmedical costs (transportation)					
	Round trip unit cost	Cost per patient for outpatient	9.0 USD	E	4)
	Round trip unit cost	Cost per patient for inpatient	22.7 USD	F	4)
	Number of visits	Per patient for outpatient	9.51	G	1)
	Number of visits	Per patient for inpatient	0.59	H	1)
	Number of patients with diabetes		4,472,133 (in base-case)	I	
	Total nonmedical cost	Total annual cost in 2019	442 million USD (in base-case)	J = (E × G + F × H) × I	
Total direct cost			13,151 million USD (in base-case)	D + J	
Indirect cost					
Caregiver’s cost					
	Care rate (inpatients employing a caregiver/total inpatients)		69.70%	K	5)
	Daily charge of a caregiver		64.8	L	5)
	Average annual inpatient days per patient due to diabetes		9.2	M	1)
	Total caregiver’s cost		1860 million USD (in base-case)	N=K × L × M	
Productivity loss cost due to morbidity					
	age		i		1)
	gender		j		1)
	Average annual hospitalization days with diabetic patients	Per patient by age and gender	Iij		1)
	Average daily income	By age and gender	Dij		6)
	Employment rates	Rates of 20–65 by age (i) and gender (g)	Pij		7)
	Average annual outpatient days with diabetic patients	Per patient by age and gender	Oij		1)
	Average hours per outpatient visit		V = 1.3		5)
	Average hourly wage	By age and gender	Hij		6)
	Total productivity loss cost due to morbidity		1461 million USD (in base-case)	Q	
Productivity loss cost due to premature mortality					
	Difference between the life expectancy of the age cohort of death and the age at the time of death	1, 2, …, n	k		8)
	Age at the time of death		t		1)
	Annual discount rate	Per patient by age and gender	r = 5%		9)
	Wage growth rate	By age and gender	ω		6)
	Number of deaths caused by diabetes	By age and gender	Nij		1)
	Average annual income	By age and gender at age at the time of death	Yij		6)
	Employment rate at the time of (t + k)	By age and gender	Pij(t + k)		7)
	Total productivity loss cost due to morbidity		1820 million USD (in base-case)	S	
Total indirect cost			5141 million USD (in base-case)	N + Q + S	

*HIRA-NPS* Health Insurance Review and Assessment Service-National Patient Sample

Q: Total productivity loss cost due to morbidity

$$ =\sum \limits_i\sum \limits_j\left\{\left({I}_{ij}\times {D}_{ij}\times {P}_{ij}\right)+\left({O}_{ij}\times V\times {H}_{ij}\times {P}_{ij}\right)\right\} $$

S: Productivity loss cost due to premature mortality

$$ ={\sum}_i{\sum}_j{\sum}_{k=1}^n\left({N}_{ij}\times \frac{Y_{ij\left(t+k\right)}\times {\left(1+\omega \right)}^k\times {P}_{ij\left(t+k\right)}}{{\left(1+r\right)}^k}\right) $$

1) HIRA-NPS claims data

2) Survey on medical expenditure of patients insured by National Health Insurance from Korea National Health Insurance Service, http://stat.kosis.kr/nsibsHtmlSvc/fileView/FileStbl/fileStblView.do?in_org_id=350&in_tbl_id=DT_350005_FILE2017&tab_yn=N&conn_path=MT_ZTITLE?in_org_id=350&in_tbl_id=DT_350005_FILE2017.

3) Survey on consumer Price Index from Korean Statistical Information Service, http://kosis.kr/statHtml/statHtml.do?orgId=101&tblId=DT_1J17001&conn_path=I2.

4) Third Korea National Health and Nutrition Examination Survey Data

5) Report of Korea Health Panel Survey, http://www.kihasa.re.kr

6) Survey on working status from Korean Statistical Information Service, http://kosis.kr/statHtml/statHtml.do?orgId=118&tblId=DT_118N_PAYM32&conn_path=I2.

7) Survey on economically active population from Korean Statistical Information Service, https://kosis.kr/statHtml/statHtml.do?orgId=101&tblId=DT_1DA7012S&conn_path=I2

8) Life table from Korean Statistical Information Service, https://kosis.kr/statHtml/statHtml.do?orgId=101&tblId=DT_1B42&conn_path=I2

9) HIRA economic evaluation guideline

### Data analysis

Descriptive statistics were calculated for several characteristics of the study population. Continuous data were presented as means and standard deviation (SD), while categorical data were shown as frequencies and percentages (%). Clinical characteristics (i.e., type of diabetes, diabetes medication use, and diabetic complications or related comorbidities) and healthcare utilization (i.e., inpatient and outpatient settings, number of outpatient visits and hospitalization, and number of hospitalization days) were examined over a 1-year period. The average annual prevalence of diabetes among the general adult population was estimated as follows. We calculated the total number of patients with diabetes using diagnostic code (E10-E14) in the 2017 samples, multiplied it by a sampling weight of 33.33% (i.e., the inverse of the sampling probability for the HIRA-NPS data), and then divided the result by the total population, using 2017 census data from the Korean Statistical Information Service [19]. Prevalence rates were stratified by age and gender. We also evaluated the economic cost of diabetes (total cost) and the average cost per patient with diabetes (per capita cost). Per capita cost was calculated as the estimated total cost divided by the number of patients with diabetes. The cost of comorbidities unrelated to diabetes (cancer, rheumatic disease, etc.) was excluded from the total cost. We analyzed diabetes-related costs beyond the primary diagnosis using attributable risk approaches to estimate more accurately the costs attributed to a disease [2]. Based on a literature review, medical claims that had a primary diagnosis of comorbidities unrelated to diabetes were excluded to prevent the enormous cost of these diseases from influencing the true diabetes-related cost [1, 20]. The specific ICD-10 codes for these diseases are listed in Additional file 1. Finally, the total cost and the total number of patients with diabetes were extrapolated to the Korean adult population by multiplying it by a sampling weight of 33.33%. All costs are presented according to each cost component, converted to U.S. dollars using an exchange rate of 1 USD to 1151 KRW, and are expressed in 2019 monetary values using the healthcare component of the Consumer Price Index for Korea [21].

We conducted subgroup analyses according to the type of diabetes, age group (< 65 vs. ≥65 years), diabetes medication use, experience of hospitalization, and presence of diabetic complications or related comorbidities. Previous studies revealed that the economic burden was affected by these variables [1, 2, 7]. The following diabetes medications were included: insulin, biguanides, sulfonylureas, thiazolidinediones, dipeptidyl peptidase-4 inhibitors, glucagon-like peptide-1 receptor agonists, meglitinides, sodium glucose cotransporter-2 inhibitors, and alpha-glucosidase inhibitors. Complications or related comorbidities often associated with diabetes were identified from the literature [1, 20] and included retinopathy, nephropathy, neuropathy, cerebrovascular disease, cardiovascular disease, peripheral vascular disease, and metabolic disease. These conditions were defined using the primary and secondary ICD-10 diagnosis codes, which are listed in Additional file 2.

## Results

### Prevalence of diabetes and characteristics of diabetes patients

**Fig. 1:**
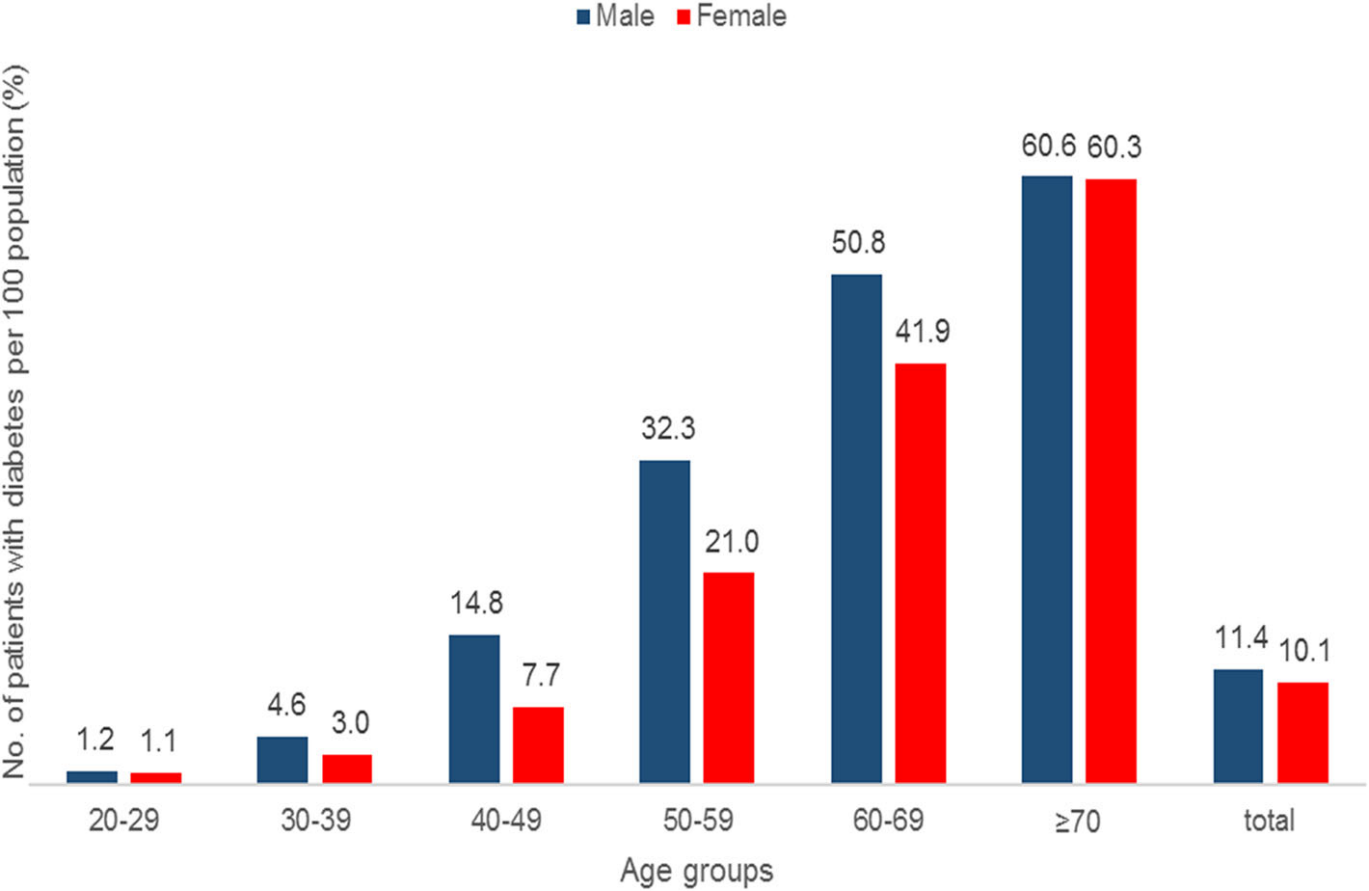
Age- and gender-specific prevalence of diabetes mellitus in Korean adults aged 20 years and older

It was estimated that a total of 4,472,133 patients were diagnosed with diabetes from the 2017 HIRA-NPS data. The average annual prevalence of diabetes in Korean adults aged 20 years and older was estimated to be 10.7%. The prevalence of diabetes was slightly higher in males (11.4%) than in females (10.1%) irrespective of the age group, which then increased gradually with age. As the patients reached their 60s, the prevalence exceeded 50% in males and the prevalence in females doubled from 21.0% in their 50s to 41.9% in their 60s (Fig. 1).

The characteristics of the study population are shown in Table 2. The study included 2,357,100 (52.7%) male and 2,115,033 (47.3%) female subjects; elderly individuals (age ≥ 65 years) accounted for 45.2% of all subjects. Most diabetes patients had type 2 diabetes (88.0%) and received diabetes medication (75.8%). Based on recorded diagnoses, 57.5% of all diabetes patients had at least one diabetic complication or related comorbidity: peripheral vascular disease (24.3%) was the related disease with the highest prevalence, followed by neuropathy (18.0%), cardiovascular disease (17.8%), and nephropathy (14.8%). The proportion of diabetes patients who had been hospitalized was 23.1%: hospitalizations through emergency department visits and outpatient visits accounted for 28.9 and 71.1%, respectively. On average, diabetes patients had 9.51 outpatient visits, 0.59 hospitalizations, and 9.16 inpatient days during the 1-year period (Table 2).

**Table 2:** Characteristics of the study population

Characteristic	N	(%)
Total no. of patients with diabetes mellitus	4,472,133	(100.0)
Gender		
Male	2,357,100	(52.7)
Female	2,115,033	(47.3)
Age		
< 65	2,449,067	(54.8)
≥ 65	2,023,067	(45.2)
Type of diabetes mellitusª		
Type 1 diabetes mellitus	172,733	(3.9)
Type 2 diabetes mellitus	3,937,200	(88.0)
Other diabetes mellitus	362,200	(8.1)
Use of diabetes medication		
One or more diabetes medications	3,391,733	(75.8)
No medication	1,080,400	(24.2)
Prevalence of diabetic complications or related comorbidities		
Retinopathy	262,933	(5.9)
Nephropathy	662,333	(14.8)
Neuropathy	806,433	(18.0)
Cerebrovascular disease	494,700	(11.1)
Cardiovascular disease	795,233	(17.8)
Peripheral vascular disease	1,088,033	(24.3)
Metabolic disease	321,533	(7.2)
Number of diabetic complications or related comorbidities		
None	1,901,667	(42.5)
1	1,569,367	(35.1)
2	727,567	(16.3)
≥ 3	273,533	(6.1)
Type of medical institution		
Primary	2,038,933	(45.6)
Secondary	1,678,800	(37.5)
Tertiary	754,400	(16.9)
Healthcare utilization^b^		
Had experience of hospitalization (inpatient setting)	1,033,267	(23.1)
Through emergency department visit	298,367	(6.7)
Through outpatient visit	734,900	(16.4)
Had no experience of hospitalization (outpatient setting)	3,438,867	(76.9)
No. of outpatient visits per patient, mean (SD)	9.51	(12.36)
No. of hospitalizations per patient, mean (SD)	0.59	(1.90)
Inpatient days per patient, mean (SD)	9.16	(42.20)

^a^ICD-10 codes to define type of diabetes mellitus: Type 1 diabetes mellitus (E10), Type 2 diabetes mellitus (E11), Malnutrition-related diabetes mellitus (E12), Other specified diabetes mellitus (E13), Unspecified diabetes mellitus (E14). Other diabetes mellitus included E12, E13, or E14. Among all diabetic patients, patients without a type 1 or type 2 diabetes code (E10 or E11) were classified as other diabetes mellitus

^b^Patients admitted as outpatients and inpatients in the same year were counted as inpatients

### Socioeconomic burden of diabetes patients

The average annual cost spent by individual patients to treat diabetes was USD 4090 in 2019 (Table 3). Direct costs were calculated to be USD 2941 per patient (71.9%) and indirect costs to be USD 1105 per patient (28.1%). The total economic burden of diabetes in South Korea was estimated to be USD 18,293 million. Medical costs accounted for the largest proportion of the total cost (69.5%), followed by productivity loss costs (17.9% = 9.9% due to premature death + 8.0% due to morbidity), caregivers’ costs (10.2%), and nonmedical costs (2.4%). While only 23.1% of the diabetes patients were hospitalized (Table 2), medical costs for inpatient services accounted for 42.7% of the total economic cost.

**Table 3:** Total annual costs in the year 2019

	Per capita cost, USD	Total cost, million USD	(%)
Direct cost	2941	13,151	(71.9)
Medical cost	2842	12,710	(69.5)
Outpatient services	1096	4904	(26.8)
Inpatient services	1745	7806	(42.7)
Nonmedical cost^a^	99	442	(2.4)
Indirect cost	1105	5141	(28.1)
Caregivers’ cost	416	1860	(10.2)
Productivity loss cost	734	3281	(17.9)
Due to morbidity	327	1461	(8.0)
Due to premature mortality	407	1820	(9.9)
Total	4090	18,293	(100.0)

Costs converted into US dollars using an exchange rate of 1 USD = 1151 KRW (2019)

^a^Nonmedical costs included transportation costs

The total annual per capita cost was 2.2 times higher (USD 8891 vs. USD 4035) for patients with type 1 diabetes than it was for those with type 2 diabetes. Particularly, indirect costs comprised a higher proportion (31.2% vs. 26.5%) for patients with type 1 diabetes than for those with type 2 diabetes (Table 4). However, the total cost was 10.3 times higher (USD 15,888 million vs. USD 1536 million) for patients with type 2 diabetes than it was for those with type 1 diabetes, which accounted for 86.9 and 8.4% of the total economic burden of the adult diabetes patients, respectively.

**Table 4:** Total annual costs according to the type of diabetes mellitus in the year 2019

	Type 1 diabetes mellitus^a^		Type 2 diabetes mellitus^a^	
	Per capita cost, USD	Total cost, million USD (%)	Per capita cost, USD	Total cost, million USD (%)
Direct cost	6117	1057 (68.8)	2968	11,684 (73.5)
Medical cost	5955	1029 (67.0)	2864	11,278 (71.0)
Nonmedical cost^a^	162	28 (1.8)	103	407 (2.6)
Indirect cost	2774	479 (31.2)	1068	4204 (26.5)
Caregiver’s cost	1062	183 (11.9)	401	1579 (9.9)
Productivity loss cost	1712	296 (19.3)	667	2625 (16.5)
Due to morbidity	865	149 (9.7)	325	1279 (8.0)
Due to premature mortality	847	146 (9.5)	342	1346 (8.5)
Total	8891	1536 (100.0)	4035	15,888 (100.0)

^a^ICD-10 codes that define the type of diabetes mellitus: Type 1 diabetes mellitus (E10), Type 2 diabetes mellitus (E11)

^b^Non-medical costs include transportation costs

**Fig. 2:**
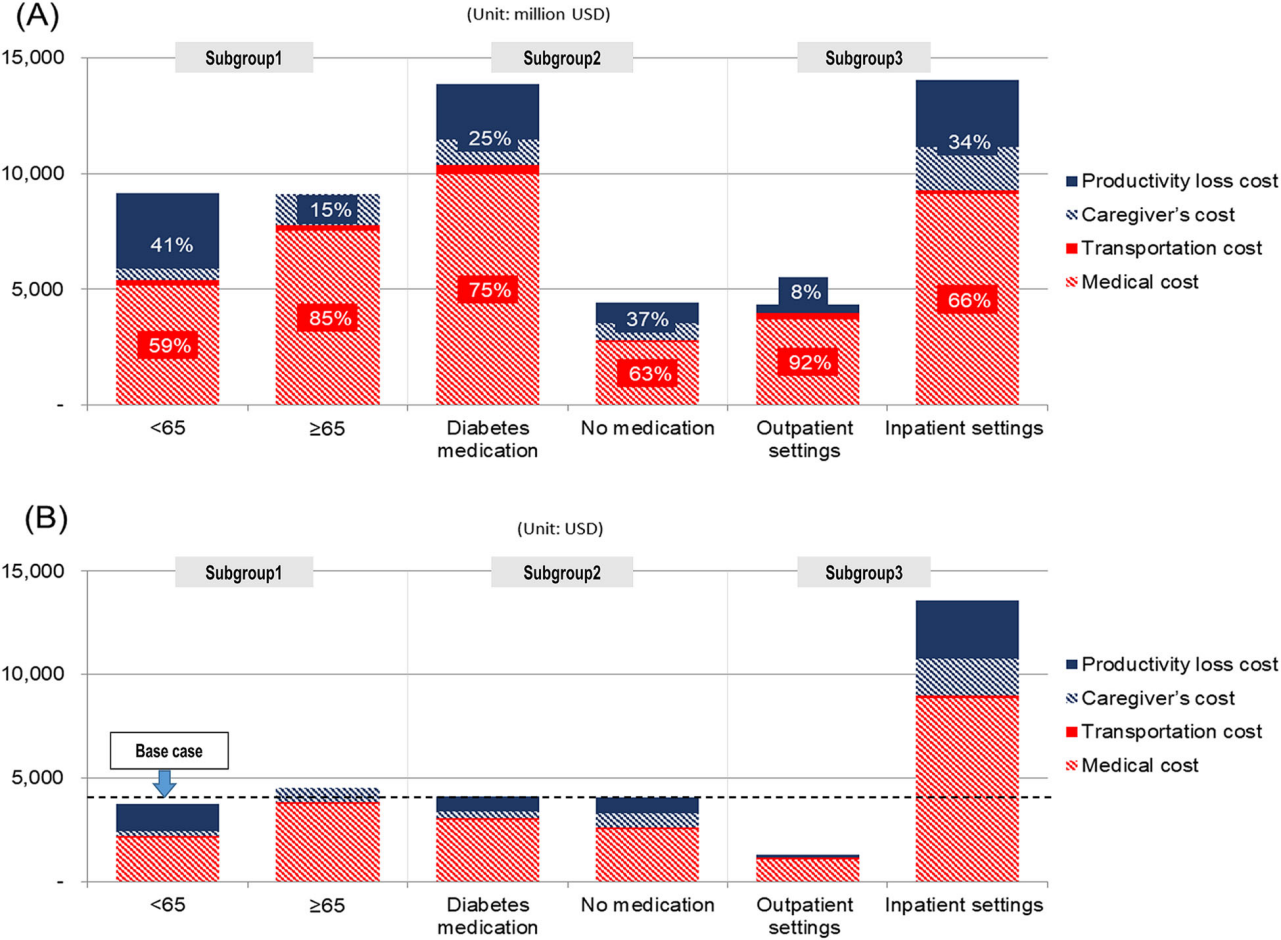
Total annual costs by subgroup in 2019. **a** Total cost (unit: million USD) according to subgroups 1–3. **b** Per capita cost (unit: USD) according to Subgroups 1–3. Per capita cost for base case (total diabetes patients) was USD 4090. subgroup 1 = age group (< 65 vs. ≥65), subgroup 2 = use of diabetes medication (medication vs. no medication), subgroup 3 = experience of hospitalization (inpatient vs. outpatient settings)

Figure 2 shows the results of the analyses by subgroup such as age group (< 65 vs. ≥65), use of diabetes medication (diabetes medication and no medication), and hospital setting (inpatient and outpatient). The annual per capita cost for adult patients over the age of 65 was higher than that for those under the age of 65, and the total cost was similar in the two groups. The per capita costs were similar for both individuals using and those not using medication; however, the total cost was higher for patients using diabetes medication than it was for those not using diabetes medication. For inpatient settings, the per capita cost was much higher (approximately 10.8 times) than that for outpatient settings: in particular, it was the highest for patients hospitalized through emergency department visits (USD 17,177, data not shown). The total cost was also higher for inpatient settings than for outpatient settings. Meanwhile, the total cost for patients treated in outpatient settings was dominated by direct costs, including medical costs (92%). For patients under the age of 65, those not using diabetes medication, and those treated in inpatient settings, indirect costs accounted for the highest proportion of total costs (41, 37, and 34%, respectively).

Adult patients with complications or related comorbidities had more outpatient visits (11.0 vs. 7.5), hospitalizations (0.8 vs. 0.3), and longer lengths of stay (12.3 vs. 5.0) for diabetes treatment in a year than did those without complications (Table 5). As the number of complications or related comorbidities per patient increased from one to three or more complications, healthcare utilization likewise increased: from 9.4 to 17.7 for outpatient visits, from 0.6 to 1.6 for hospital admission, and from 9.1 to 24.9 for length of stay. In particular, patients with three or more complications had approximately five times as many average numbers of hospitalizations and five times as long average lengths of stay as patients without complications. Further, as the number of complications or related comorbidities increased from one to three or more, the per capita cost of diabetes treatment increased from USD 3991 to USD 11,965. The total cost was higher (USD 14,176 million vs. USD 4117 million) for patients with complications than for those without complications, which accounted for 77.5 and 22.5% of the total economic burden among adult diabetes patients, respectively.

**Table 5:** Healthcare utilization and total annual costs for diabetes patients with diabetic complications or related comorbidities

	No complication		With complications or related comorbidities							
			Total		One complication		Two complications		Three or more complications	
Total no. of patients with diabetes mellitus, n (%)	1,901,667	(42.5)	2,570,467	(57.5)	1,569,367	(35.1)	727,567	(16.3)	273,533	(6.1)
No. of outpatient visits per patient, mean (SD)	7.5	(6.5)	11.0	(15.2)	9.4	(9.9)	11.9	(16.2)	17.7	(28.9)
No. of hospitalizations per patient, mean (SD)	0.3	(1.4)	0.8	(2.2)	0.6	(1.9)	0.9	(2.4)	1.6	(3.2)
Duration of hospitalization per patient, days, mean (SD)	5.0	(32.2)	12.3	(48.0)	9.1	(42.4)	14.4	(51.4)	24.9	(63.9)
Per capita cost, USD	2159		5499		3991		6289		11,965	
Total cost, million USD	4117		14,176		6291		4597		3288	

Costs converted into US dollars using an exchange rate of 1 USD = 1151 KRW (2019)

## Discussion

This is the first nationwide study to estimate the prevalence and socioeconomic burden of diabetes using a nationally representative claims database in South Korea. This study showed that the prevalence of diabetes in Korean adults was 10.7%. The diabetes-related economic burden was USD 18,293 million, with an average per capita cost of USD 4090 in 2019. Our results also showed that type 2 diabetes, presence of diabetic complications or related comorbidities, diabetes medication use, and hospitalization were associated with a large economic burden of diabetes.

The estimated prevalence of diabetes in Korean adults was slightly higher than the global prevalence of diabetes in the adult population (8.5%) [3]. According to the Korean National Health and Nutrition Examination Survey (2007–2009), 11.0% of males and 8.9% of females among the adult population had diabetes [10]. These results are similar to ours (11.4% in males and 10.1% in females) considering the increasing trend in the prevalence of diabetes.

The economic burden of diabetes was USD 18,293 million in South Korea in 2019, which is equivalent to approximately 1.14% of Korea’s gross domestic product (GDP). A study published in the U.S. showed that the estimated national cost of diabetes in 2017 (USD 327 billion) accounted for 1.69% of the GDP [1]. It could be said that the relatively high percentage of GDP in the U.S. is caused by the high per capita cost for diabetes (USD 16,752), especially considering that the prevalence of diabetes in the U.S. is slightly lower (9.7% of the adult population) than it is in Korea. Additionally, the total annual cost of major diseases in Korea, such as cancer [22], liver disease [23], and cardio-cerebrovascular disease [24, 25], has been reported in the range of USD 1 billion to 3 billion, which was much lower than the total cost of diabetes. Moreover, the economic burden of diabetes was higher than the economic burden for overall cancers, at USD 15 billion [22].

Type 2 diabetes is the most common type of diabetes, accounting for approximately 90% of all cases of diabetes [6]. Our study showed that 88.0% cases of all types of diabetes were type 2, which also accounted for most of the economic burden associated with diabetes (86.9%). In Korea, the direct medical costs of type 2 diabetes corresponded to 10.6% of all healthcare expenditure (USD 85.5 billion, calculated as only direct medical costs excluding out-of-pocket costs [26]). This was higher than the cost of type 2 diabetes in France, where it corresponded to ~ 5% of all healthcare expenditures [27]. The economic burden makes type 2 diabetes a major clinical and public health problem in Korea [2]. Type 2 diabetes is associated with being overweight and obese. Approximately half (48.6%) of adult patients with diabetes are obese in Korea [28]. Therefore, future efforts to reduce the global health and economic burden of diabetes should emphasize the prevention of type 2 diabetes, or delaying its onset, by promoting healthy behavior and diet at the population level [11].

As with most other diseases, elderly patients require more healthcare resources to treat diabetes than younger patients. In this study, approximately half of all health care expenditure related to diabetes were spent by patients over the age of 65. They spent about 1.2 times more in annual per capita cost than patients under the age of 65. High medical expenditures among the elderly, along with high caregivers’ costs related to hospitalization, can be partly attributed to the increased risk of hospitalization that comes with aging. The hospitalization rate of those over the age of 65 among our study subjects was approximately 1.5 times higher than that of those under the age of 65 (28.2% vs. 18.9%). These results suggest the need for active interventions for the elderly. For example, workplace health promotions that target older workers or mobile health programs to facilitate adherence of patients to chronic disease management can improve health outcomes and quality of life for diabetics and reduce the economic burden of the disease [29, 30]. On the one hand, early management for prevention for those under the age of 65 years can reduce the burden of diabetes. In particular, increased levels of obesity and physical inactivity among young people, can potentially lead to serious health outcomes with high medical expenditures in old age; therefore, prevention strategies are required to control these modifiable risk factors. Lifestyle interventions for young and middle-aged people can be implemented to prevent long-term diabetes complications [31].

We confirmed that hospitalization was a cost driver, which is associated with high costs for diabetes [2]. Inpatient services accounted for 42.7% of the total economic costs for all diabetes patients. Diabetes incurred higher spending for inpatient services than other diseases that are common among the elderly, such as hypertension (18.3%), rheumatic arthritis (7.9%), heart failure (29.2%), and asthma (11.7%) [32–34]. Furthermore, the total costs for diabetes patients with an experience of hospitalization represented 76.4% of the total economic costs for all diabetes patients, and the per capita cost in inpatient settings was much higher than that in outpatient settings, by 10.8 times. Our results showed that inpatients represented a higher percentage of the elderly (55% vs. 42%) and those with complications (71.1% vs. 53.4%) than did outpatients. Patients requiring hospitalization generally had severe conditions, and because of the high cost of premature deaths from such conditions, indirect costs for inpatients accounted for a higher proportion of total costs (34% vs. 8%) than for outpatients. These findings suggest that an effective intervention to prevent the progression to severe conditions that require hospitalization should be a critical component of a disease management strategy to minimize the economic and clinical burden of diabetes. For example, patients with diabetes should implement self-management strategies such as healthy lifestyle choices, and education for strict blood glucose and blood pressure management; these efforts are essential to reduce the risk of development and progression of diabetes complications [6].

Because most patients with diabetes (75.8%) have been prescribed diabetes drugs, the total economic burden of patients using diabetes medication was high. However, per capita cost was similar between patients using diabetes medication and those with no use of diabetes medication, and indirect costs accounted for a higher proportion of the total for patients with no use of diabetes medication (37%) because of the higher costs incurred by diabetes complications and premature deaths than for those using diabetes medication (25%). Continuous treatment of diabetes is particularly important for preventing diabetes-related complications [35]. Large cohort studies found that the improvement of anti-diabetic medication adherence among diabetes patients significantly decreased the risk of macrovascular or microvascular complications s [36, 37]. Therefore, receiving medication may reduce the clinical burden at the individual level, and also reduce the socioeconomic burden by reducing indirect costs at the population level.

Our study found that per capita costs of diabetes increased with the number of complications or related comorbidities. As the number of complications increased, hospitalization also increased; in particular, the percentage of hospitalizations through emergency departments increased from 19.8 to 43.6% (data not shown). This result is in line with that of other studies that showed that medical costs incurred by complications led to a high economic burden in diabetic patients [7, 38]. The American Diabetes Association reported how diabetes contributed to the direct medical cost of major complications: the proportion of expenditures attributed to diabetes for peripheral vascular, neurological, renal, and cardiovascular diseases over total U.S. health care expenditures (39, 36, 29, and 27%, respectively) was higher than that for other general medical conditions (8%) [1]. We confirmed that peripheral vascular disease was the most common complication in adult diabetic patients in Korea, followed by neurological, cardiovascular, and renal diseases. Moreover, among diabetes complications or related comorbidities, cardiovascular, peripheral vascular, and renal diseases had the most expensive direct medical costs (Additional file 3). For early detection of these complications, screening tests are important parts of successful prevention or delay of diabetic complications, and diabetes self-management education has proven effective in reducing harmful and costly complications in high-income countries worldwide [6, 39]. The prevention and management of these complications can significantly reduce not only diabetes, but also the economic burden of this disease.

This study had several limitations. First, study subjects with diabetes were identified only based on ICD-10 codes, which potentially allowed misclassification or miscoding. Because the HIRA-NPS data do not provide information on laboratory test parameters such as fasting plasma glucose, oral glucose tolerance, and HbA1c levels, we were not able to confirm diabetes cases based on diagnostic test results. However, a previous study indicated that diabetes could be accurately identified in administrative data: the definition of diabetes, 2 physician claims within 1 years or 1 hospitalization with the ICD-10 codes E10.x–E14.x, had high validity (sensitivity 91.6%, specificity 97.2%) [13]. Therefore, we consider that the administrative data can be used to establish the population-based prevalence of diabetes as a reasonable alternative to biochemical assay data [40]. Second, health insurance claims data did not include information about subjects with undiagnosed or untreated diabetes. It was estimated that over 50% of adults with diabetes in the Western Pacific region were undiagnosed according to the IDF Diabetes Atlas [5]. Won et al. (2018) reported that the estimated prevalence of undiagnosed or diagnosed diabetes was 13.7% during 2013–2014 in Korean adults (≥30 years of age) [28], which was higher than the published prevalence of diagnosed diabetes [10]. In addition, among patients receiving diabetes medication in Korea, the visiting rate to medical institutions within 1 year from the first visit was 94%, and rate of medication adherence was 57.8%, not high [41]. Therefore, the prevalence as well as the cost of diabetes might be underestimated. However, our study was conducted among diagnosed patients who had paid for healthcare service using very comprehensive health care claims data that cover the nationwide Korean population. We regard our results to be conservative in terms of estimating COI and proper as being representative for the prevalence of diabetes in South Korea. Third, cross-sectional studies using claims data make it difficult to identify causal relationships between diabetes and its complications or related comorbidities. Thus, healthcare resources use by diabetes patients with related complications can be overestimated. To reduce the potential of overestimation in our definition of cases with complications, we excluded those for whom diabetes and complications were not diagnosed in the same prescription, although complications may occur within a certain period after the initial diagnosis of diabetes. Additionally, we mainly used diabetic complication codes (such as E10.1-E10.5 to E14.1–14.5, Additional file 2) to increase the association with diabetes, and all codes defining complications or related comorbidities were validated [1, 20]. The known prevalence of diabetic complications or related comorbidities varies from one country to another; the prevalence of diabetic complications in Korea was similar or slightly lower than the average prevalence globally [6]. Despite these limitations, we believe that the administrative data used in this study provide a powerful resource for a population-based evaluation of the economic burden of diabetes [40].

## Conclusions

The study showed that in South Korea the prevalence of diabetes in adults was slightly higher than the global prevalence, and the economic burden of diabetes was higher than that of the overall cancer. In particular, we confirmed that diabetic complications or related comorbidities and hospitalization were associated with high costs for diabetes as a cost driver. These findings highlight the need for effective strategies to manage patients with diabetic complications to reduce the use of healthcare resources and economic burden. It is expected that increased information regarding both the magnitude and the specific components of the economic burden of diabetes in Korea will influence health policy makers to prioritize its prevention and management and to allocate more health care resources to diabetes.

## Supplementary Information

**Additional file 1.** ICD-10 codes that define comorbidities unrelated to diabetes.

**Additional file 2.** ICD-10 codes that define diabetic complications and related comorbidities.

**Additional file 3.** Direct medical costs by diabetic complications and related comorbidities.

## Data Availability

The data that support the findings of this study are available from the Health Insurance Review and Assessment Service but restrictions apply to the availability of these data, which were used under license for the current study, and so are not publicly available. Data are however available from the authors upon reasonable request and with permission of the Health Insurance Review and Assessment Service.

## Abbreviations

COI: Cost-of-illness
IDF: International Diabetes Federation
HIRA-NPS: Health Insurance Review and Assessment Service National Patient Sample
ICD-10: International Classification of Diseases Codes, 10th revision
SD: Standard deviation
GDP: Gross domestic product

## References

[1] American DA (2018). Economic costs of diabetes in the U.S. in 2017. Diabetes Care.

[2] Ettaro L, Songer TJ, Zhang P, Engelgau MM (2004). Cost-of-illness studies in diabetes mellitus. Pharmacoeconomics..

[3] World Health Organization. Global report on diabetes. World Health Organization. 2016. Available from: https://www.who.int/publications/i/item/9789241565257. Accessed 1 Oct 2019.

[4] Seuring T, Archangelidi O, Suhrcke M (2015). The economic costs of type 2 diabetes: a global systematic review. Pharmacoeconomics..

[5] Ogurtsova K, da Rocha Fernandes JD, Huang Y, Linnenkamp U, Guariguata L, Cho NH (2017). IDF diabetes atlas: global estimates for the prevalence of diabetes for 2015 and 2040. Diabetes Res Clin Pract.

[6] International Diabetes Federation (2017). IDF Diabetes Atlas eighth edition.

[7] Huang Y, Vemer P, Zhu J, Postma MJ, Chen W (2016). Economic burden in Chinese patients with diabetes mellitus using electronic insurance claims data. Plos One.

[8] Fernandes S, Fernandes S (2017). Economic burden of diabetes mellitus and its socio-economic impact on household expenditure in an urban slum area. Int J Res Med Sci.

[9] Pagano E, Brunetti M, Tediosi F, Garattini L (1999). Costs of diabetes. A methodological analysis of the literature. Pharmacoeconomics..

[10] Kim DJ (2011). The epidemiology of diabetes in Korea. Diabetes Metab J.

[11] NCD-RisC (2016). Worldwide trends in diabetes since 1980: a pooled analysis of 751 population-based studies with 4.4 million participants. Lancet.

[12] Kim L, Kim JA, Kim S (2014). A guide for the utilization of Health Insurance Review and Assessment Service national patient samples. Epidemiol Health.

[13] Chen G, Khan N, Walker R, Quan H (2010). Validating ICD coding algorithms for diabetes mellitus from administrative data. Diabetes Res Clin Pract.

[14] Scalone L, Cesana G, Furneri G, Ciampichini R, Beck-Peccoz P, Chiodini V (2014). Burden of diabetes mellitus estimated with a longitudinal population-based study using administrative databases. Plos One.

[15] Survey on medical expenditure of patients insured by National Health Insurance. Korea National Health Insurance Service. 2018. Available from: http://stat.kosis.kr/nsibsHtmlSvc/fileView/FileStbl/fileStblView.do?in_org_id=350&in_tbl_id=DT_350005_FILE2017&tab_yn=N&conn_path=MT_ZTITLE?in_org_id=350&in_tbl_id=DT_350005_FILE2017. Accessed 1 Oct 2019.

[16] The Third Korea National Health and Nutrition Examination Survey. Korean Ministry of Health and Welfare Affairs. 2006.

[17] Lofland JH, Pizzi L, Frick KD (2004). A review of health-related workplace productivity loss instruments. Pharmacoeconomics..

[18] Survey on working status. Korean Statistical Information Service. 2017. Available from: http://kosis.kr/statHtml/statHtml.do?orgId=118&tblId=DT_118N_PAYM32&conn_path=I2. Accessed 1 Oct 2019.

[19] Population census data. Korean Statistical Information Service. 2017. Available from: http://kosis.kr/statHtml/statHtml.do?orgId=101&tblId=DT_1BPA001&conn_path=I2. Accessed 1 Oct 2019.

[20] Chang HY, Weiner JP, Richards TM, Bleich SN, Segal JB (2012). Validating the adapted diabetes complications severity index in claims data. Am J Manag Care.

[21] Consumer Price Index. Korean Statistical Information Service. 2019. Available from: http://kosis.kr/statHtml/statHtml.do?orgId=101&tblId=DT_1J17001&conn_path=I2. Accessed 1 Oct 2019.

[22] Kim J, Hahm MI, Park EC, Park JH, Park JH, Kim SE (2009). Economic burden of cancer in South Korea for the year 2005. J Prev Med Public Health.

[23] Jung YH, Ko S (2006). The socioeconomic cost of diseases in Korea. J Prev Med Public Health.

[24] Lim SJ, Kim HJ, Nam CM, Chang HS, Jang YH, Kim S (2009). Socioeconomic costs of stroke in Korea: estimated from the Korea national health insurance claims database. J Prev Med Public Health.

[25] Chang HS, Kim HJ, Nam CM, Lim SJ, Jang YH, Kim S (2012). The socioeconomic burden of coronary heart disease in Korea. J Prev Med Public Health.

[26] Healthcare Bigdata Hub. Health Insurance Review & Assessment Service. 2019. Available from: http://opendata.hira.or.kr/op/opc/olapJdgeChargeInfo.do. Accessed 1 Oct 2019.

[27] Charbonnel B, Simon D, Dallongeville J, Bureau I, Dejager S, Levy-Bachelot L (2018). Direct medical costs of type 2 diabetes in France: an insurance claims database analysis. Pharmacoecon Open.

[28] Won JC, Lee JH, Kim JH, Kang ES, Won KC, Kim DJ (2018). Diabetes fact sheet in Korea, 2016: an appraisal of current status. Diabetes Metab J.

[29] Poscia A, Moscato U, La Milia DI, Milovanovic S, Stojanovic J, Borghini A (2016). Workplace health promotion for older workers: a systematic literature review. BMC Health Serv Res.

[30] Hamine S, Gerth-Guyette E, Faulx D, Green BB, Ginsburg AS (2015). Impact of mHealth chronic disease management on treatment adherence and patient outcomes: a systematic review. J Med Internet Res.

[31] International Diabetes Federation (2013). IDF Diabetes Atlas sixth edition.

[32] Lee H, Oh SH, Cho H, Cho HJ, Kang HY (2016). Prevalence and socio-economic burden of heart failure in an aging society of South Korea. BMC Cardiovasc Disord.

[33] Lee YJ, Kwon SH, Hong SH, Nam JH, Song HJ, Lee JS (2017). Health care utilization and direct costs in mild, moderate, and severe adult asthma: a descriptive study using the 2014 south Korean health insurance database. Clin Ther.

[34] Fautrel B, Clarke AE, Guillemin F, Adam V, St-Pierre Y, Panaritis T (2007). Costs of rheumatoid arthritis: new estimates from the human capital method and comparison to the willingness-to-pay method. Med Decis Mak.

[35] Effect of intensive diabetes management on macrovascular events and risk factors in the Diabetes Control and Complications Trial. Am J Cardiol. 1995;75(14):894–903. 10.1016/s0002-9149(99)80683-3 7732997

[36] Kim YY, Lee JS, Kang HJ, Park SM (2018). Effect of medication adherence on long-term all-cause-mortality and hospitalization for cardiovascular disease in 65,067 newly diagnosed type 2 diabetes patients. Sci Rep.

[37] Simpson SH, Lin M, Eurich DT (2016). Medication adherence affects risk of new diabetes complications: a cohort study. Ann Pharmacother.

[38] Hu H, Sawhney M, Shi L, Duan S, Yu Y, Wu Z (2015). A systematic review of the direct economic burden of type 2 diabetes in China. Diab Ther.

[39] Clark M (2008). Diabetes self-management education: a review of published studies. Prim Care Diabetes.

[40] Hux JE, Ivis F, Flintoft V, Bica A (2002). Diabetes in Ontario: determination of prevalence and incidence using a validated administrative data algorithm. Diabetes Care.

[41] Hyeongsu K, Soon-Ae S, Kunsei L, Jong-Heon P, Tae Hwa H, Minsu P (2018). Effects of first diagnosed diabetes mellitus on medical visits and medication adherence in Korea. Iran J Public Health.

