# Supplementary material for: Prevalence and socioeconomic burden of diabetes mellitus in South Korean adults: a population-based study using administrative data
Source: BMC Public Health. 2021 Mar 20;21:548. doi: 10.1186/s12889-021-10450-3 (PMC7980668; doi:10.1186/s12889-021-10450-3)
Supplement: Supplementary file 1 — Additional file 1. ICD-10 codes that define comorbidities unrelated to diabetes. [file 12889_2021_10450_MOESM1_ESM.docx]

Additional File 1. ICD-10 codes that define comorbidities unrelated to diabetes

|  | ICD-10 codes |
| --- | --- |
| COPD | I27.8, I27.9, J40.x–J47.x, J60.x–J67.x, J68.4, J70.1, J70.3 |
| Rheumatic disease | M05.x, M06.x, M31.5, M32.x–M34.x, M35.1, M35.3, M36.0 |
| Peptic ulcer disease | K25.x–K28.x |
| Cancer^a^ | C00.x–C26.x, C30.x–C34.x, C37.x–C41.x, C43.x, C45.x–C58.x, C60.x–C76.x, C81.x–C85.x, C88.x, C90.x–C97.x |
| Metastatic solid tumor | C77.x–C80.x |
| ^a^Cancer included leukemia, malignant lymphoma, and solid tumor.  COPD, Chronic obstructive pulmonary disease; ICD-10, International Classification of Diseases, 10th revision | |
