# Supplementary material for: Prevalence and socioeconomic burden of diabetes mellitus in South Korean adults: a population-based study using administrative data
Source: BMC Public Health. 2021 Mar 20;21:548. doi: 10.1186/s12889-021-10450-3 (PMC7980668; doi:10.1186/s12889-021-10450-3)
Supplement: Supplementary file 2 — Additional file 2. ICD-10 codes that define diabetic complications and related comorbidities. [file 12889_2021_10450_MOESM2_ESM.docx]

Additional File 2. ICD-10 codes that define diabetic complications and related comorbidities

|  | ICD-10 codes |
| --- | --- |
| Retinopathy | E10.3, E11.3, E12.3, E13.3, E14.3, H25, H26, H28.0, H31.1, H33, H34, H35, H36, H40, H42, H43.1, H45.0, H53 |
| Nephropathy | N00-N08, N17-N19, N25-N26, E10.2, E11.2, E12.2, E13.2, E14.2, Z94.0, Z99.2 |
| Neuropathy | E10.4, E11.4, E12.4, E13.4, E14.4, G56.9, G57.9, G58.9, G59.0, G63.2, G73.3, G99.0, S04.1, S04.2, S04.4, M14.6 |
| Cerebrovascular disease | I60-I69, G45 |
| Cardiovascular disease | I20-I25, I48, I49.0, I50 |
| Peripheral vascular disease | E10.5, E11.5, E12.5, E13.5, E14.5, I70-I74, I77, I79, I82, I83 |
| Metabolic disease | E10.0, E11.0, E12.0, E13.0, E14.0, E10.1, E11.1, E12.1, E13.1, E14.1, E87 |
| ICD-10, International Classification of Diseases, 10th revision | |
