# Supplementary material for: Prevalence and socioeconomic burden of diabetes mellitus in South Korean adults: a population-based study using administrative data
Source: BMC Public Health. 2021 Mar 20;21:548. doi: 10.1186/s12889-021-10450-3 (PMC7980668; doi:10.1186/s12889-021-10450-3)
Supplement: Supplementary file 3 — Additional file 3. Direct medical costs by diabetic complications and related comorbidities. [file 12889_2021_10450_MOESM3_ESM.docx]

Additional File 3. Direct medical costs by diabetic complications and related comorbidities

|  | Total | | Hospitalization through outpatient visit | | Hospitalization through emergency department visit | | Outpatient visit | |
| --- | --- | --- | --- | --- | --- | --- | --- | --- |
|  | Per capita cost, USD | Total cost, million USD　(%) | Per capita cost, USD | Total cost, million USD　(%) | Per capita cost, USD | Total cost, million USD　(%) | Per capita cost, USD | Total cost, million USD　(%) |
| Overall | 2,842 | 12,710 (100.0%) | 7,424 | 5,456 (42.9%) | 12,065 | 3,600 (28.3%) | 1,063 | 3,654 (28.7%) |
| No complications | 1,400 | 2,663 (20.9%) | 4,912 | 1,178 (44.3%) | 6,170 | 365 (13.7%) | 698 | 1,119 (42.0%) |
| With complications | 3,909 | 10,047 (79.1%) | 8,642 | 4,278 (42.6%) | 13,525 | 3,234 (32.2%) | 1,381 | 2,535 (25.2%) |
| Retinopathy | 5,495 | 1,445 (11.4%) | 10,095 | 620 (42.9%) | 17,287 | 543 (37.6%) | 1,660 | 283 (19.6%) |
| Nephropathy | 5,445 | 3,607 (28.4%) | 10,498 | 1,242 (34.4%) | 17,905 | 1,470 (40.8%) | 1,937 | 895 (24.8%) |
| Neuropathy | 4,411 | 3,557 (28.0%) | 8,778 | 1,421 (39.9%) | 16,348 | 1,186 (33.3%) | 1,662 | 951 (26.7%) |
| Cerebrovascular disease | 7,234 | 3,579 (28.2%) | 12,220 | 1,739 (48.6%) | 15,767 | 1,406 (39.3%) | 1,645 | 433 (12.1%) |
| Cardiovascular disease | 5,520 | 4,390 (34.5%) | 9,236 | 1,796 (40.9%) | 15,040 | 1,826 (41.6%) | 1,601 | 768 (17.5%) |
| Peripheral vascular disease | 3,646 | 3,967 (31.2%) | 8,204 | 1,527 (38.5%) | 15,274 | 1,264 (31.9%) | 1,435 | 1,176 (29.6%) |
| Metabolic disease | 9,074 | 2,917 (23.0%) | 10,767 | 1,138 (39.0%) | 18,348 | 1,472 (50.5%) | 2,268 | 308 (10.5%) |
